# From Population-Based PBPK to Individualized Virtual Twins: Clinical Validation and Applications in Medicine

**DOI:** 10.3390/jcm15031210

**Published:** 2026-02-04

**Authors:** Marta Gonçalves, Pedro Barata, Nuno Vale

**Affiliations:** 1 PerMed Research Group, RISE-Health, Faculty of Medicine, University of Porto, Alameda Professor Hernâni Monteiro, 4200-319 Porto, Portugal; up202005255@edu.med.up.pt; 2 Laboratory of Personalized Medicine, Department of Community Medicine, Health Information and Decision (MEDCIDS), Faculty of Medicine, University of Porto, Rua Doutor Plácido da Costa, 4200-450 Porto, Portugal; 3 Faculty of Health Sciences, University of Fernando Pessoa, Rua Carlos da Maia, 296, 4200-150 Porto, Portugal; pbarata@ufp.edu.pt; 4 RISE-Health, Faculty of Health Sciences, University of Fernando Pessoa, Praça 9 de Abril, 349, 4249-004 Porto, Portugal; 5 Unidade Local de Saúde de Santo António, E.P.E., Largo Professor Abel Salazar, 4099-001 Porto, Portugal; 6 RISE-Health, Department of Community Medicine, Health Information and Decision (MEDCIDS), Faculty of Medicine, University of Porto, Rua Doutor Plácido da Costa, 4200-450 Porto, Portugal

**Keywords:** PBPK, personalized medicine, model performance, clinical validation

## Abstract

Physiologically based pharmacokinetic (PBPK) models are widely used in the context of personalized medicine, as they allow for the evaluation of dosing schedules and routes of administration by predicting absorption, distribution, metabolism and excretion (ADME) of drugs in biological systems. Traditionally, PBPK models have been developed and applied at the population level, enabling the characterization of predefined cohorts, which remains limited in supporting true precision dosing. In this review, we explored the increasingly common shift from population-based to individual PBPK modelling, where individuals are modelled as virtual twins (VTs). Through the inclusion of additional patient-specific data, such as demographic, physiological, phenotypic and genotypic information, models can be personalized, moving beyond traditional one-size-fits-all strategies. Overall, incorporating individual patient data (e.g., septic, psychiatric, cardiac, or neonatal populations) improves model performance. Physiological parameters, particularly renal function, show strong potential given their role in drug elimination, while demographic variables enhance predictive accuracy in certain studies. In contrast, the benefits of including cytochrome P450 (CYP) phenotypic and genotypic data remain inconsistent. We further emphasize methodologies used to evaluate model performance, with a focus on clinical validation through comparisons between predicted and observed concentration-time profiles. Key challenges, including limited sample sizes and data availability, that may compromise predictive precision, are also discussed. Finally, we highlight the potential integration of PBPK-based VTs into broader digital twin frameworks as a promising path toward clinical translation, while acknowledging the critical barriers that must be addressed to enable routine clinical implementation.

## 1. Introduction

Historically, the inherent physiological complexity of the human body compelled researchers to employ simplified models to predict dynamics, including exponential decay and compartment models, which had limited power to govern pharmacokinetic behaviour of drugs in tissues and organs. These oversimplifications have, in turn, been associated with increased rates of drug attrition [1]. Nevertheless, the emergence of mechanistic and systems-based modelling approaches has enabled a more comprehensive and biologically grounded description of drug behaviour in vivo. Modelling in this context encompasses four essential phases: construction, validation, learning, in which deviations are uncovered and interpreted within a solid scientific framework, and refinement, where the newly acquired knowledge is integrated to enhance model performance [2]. Within biomedical research, modelling has therefore emerged as a powerful and integrative approach. By uncovering interaction effects embedded in complex datasets that are often inaccessible to human intuition, models facilitate mechanistic insight, enable hypothesis generation and testing, support outcome prediction, and inform experimental design. Furthermore, modelling can reduce development costs, limit the need for extensive animal experimentation, and increase the efficiency of hypothesis validation [3].

Traditionally, therapeutic drug monitoring (TDM) has been used to support dose individualisation by measuring drug concentration after administration, rendering it an inherently retrospective approach. Physiologically based pharmacokinetic (PBPK) models complement TDM by integrating physiological and clinical data to prospectively predict drug exposure, enabling model-informed precision dosing (MIPD).

PBPK models are a mechanistic subgroup within the quantitative systems pharmacology (QSP) models. These models allow researchers to connect the external exposure of a chemical with its absorption, leading to measurable values of time-dependent concentrations in organs and tissues. Among QSP models, PBPK is an example of one that uses ordinary differential equations and aims to represent that is physiologically accurate of organs and tissues [4]. They achieve this by integrating drug-specific properties with independent knowledge of the organism’s physiology and biology [5]. To fully grasp the principles underlying PBPK modelling, it is essential to first understand the fundamental concept of pharmacokinetics. This concept integrates the series of processes that govern the fate of a drug within the body, namely absorption, distribution, metabolism and excretion (ADME) [6,7]. PBPK models, which are typically calibrated using pharmacokinetic data obtained from a single dose regimen, can subsequently be used to evaluate alternative dosing schedules and routes of administration [8].

Over the past decade, PBPK models have become increasingly important in drug development and regulatory science, where they are used, for instance, to assess drug–drug interactions and to predict drug exposure and response in special populations, such as children or organ impairment patients [9,10]. Regulatory agencies, including the Food and Drug Administration (FDA), now accept PKPB analyses in submissions [11].

Key challenges in classical PBPK modelling include accurately representing uncertainty and interindividual variability. These challenges arise from decisions about model structure, the integration of deterministic and probabilistic components, and assumptions about physiological similarity across populations. Additional difficulties relate to parameter calibration, including distinguishing between model fitting and validation, identifying relevant sources of variability, and integrating heterogeneous data [12]. Classical population PBPK approaches aim to approximate interindividual physiological variability through virtual populations. However, this does not fully capture the true extent of between-subject differences that drive individual drug exposure [13]. These general models support group-based dosing, while remaining limited by their reliance on average physiology and assumptions that may not fully reflect individual variability. On the other hand, individual MIPD provides a more precise and clinically relevant approach to dose optimization, by contradicting classical one-size-fits-all approaches, through the incorporation of patient-specific data and adaptive model updating [14]. Back in 2017, Tucker G. discussed the potential of associating an individual patient with a corresponding virtual twin (VT), identifying this concept as a future challenge in modelling [15]. Since then, significant methodological advances have been achieved, and, within the MIPD framework, VTs represent a novel paradigm in which individualized PBPK models are constructed to reflect patient-specific physiological and clinical characteristics, shifting the focus from a drug-centred to a patient-centred perspective [16]. In recent years, digital twins may be viewed as a possible conceptual extension of VT frameworks, enabling continuous and bidirectional integration of real-world patient data. However, their application in the context of PBPK modelling remains largely prospective.

Despite their mechanistic nature, the clinical reliability of PBPK models critically depends on rigorous validation strategies. In scientific literature, model evaluation follows distinct approaches. Some studies rely on external validation by comparing model predictions with pharmacokinetic data previously published in independent clinical studies [17], whereas others assess model performance with data obtained within the same study, by directly comparing predicted and observed concentration-time profiles of the evaluated drug [18]. This latter approach represents a clinically meaningful strategy to assess predictive performance at the individual level, ensuring model reliability, supporting dose optimization, and aiding in mitigation of the risks of false prediction. While substantial methodological advances have been made, important conceptual and practical questions in PBPK modelling remain unresolved. In particular, it is unclear how PBPK models should be individualized in a clinically robust manner, how different personalization strategies compare with classical population-based approaches, and how individualized model predictions should be evaluated to ensure clinical reliability. These unresolved issues currently limit the routine adoption of individualized PBPK models in clinical decision-making.

In this context, and given the growing interest in personalized medicine, model-informed precision dosing, and PBPK modelling, this review aims to critically examine the conceptual and methodological foundations of individualized PBPK approaches. Specifically, it focuses on strategies for personalizing PBPK models using patient-specific demographic, physiological, phenotypic, and genotypic data, as well as on validation frameworks designed to assess predictive performance at the individual level. By synthesizing current methodologies, identifying key limitations, and highlighting unresolved challenges, this review seeks to clarify the role of individualized PBPK models in supporting patient-specific clinical decision-making.

## 2. Literature Search and Selection

In this review, a structured literature search was conducted to identify studies addressing the development, individualisation, and clinical validation of PBPK models, with particular emphasis on individual-level applications framed as VTs. Electronic databases searched include PubMed, Scopus, Web of Science and Google Scholar. Only peer-reviewed articles written in English were considered. The retrieved literature was used for both conceptual framing and analytical discussion within this review. Conceptual sources were selected to support definitions, terminology, and background principles related to PBPK modelling, personalized medicine, and VT frameworks, without the application of restrictive inclusion criteria. In contrast, a focused subset of studies was selected for in-depth analytical discussion, specifically to evaluate how PBPK models have been individualized and clinically assessed at the level of real patients. For this analytical component, studies were required to report the construction of individualized PBPK models anchored to identifiable individuals and to include a direct comparison between model-predicted concentration–time profiles and drug concentrations measured in the same patient. These criteria were applied to ensure that the reviewed studies addressed clinically meaningful validation rather than population-level extrapolation.

Within this review, a PBPK VT was defined as an individual-level mechanistic PBPK model constructed to represent a single real patient, using patient-specific clinical and demographic information as model inputs, and evaluated by comparison of model-predicted concentration–time profiles with drug concentrations measured in that same patient. Literature-based or default physiological parameters were permitted when patient-specific measurements were unavailable, provided that the model remained anchored to an identifiable individual rather than a population or subgroup.

Data extracted from eligible studies included clinical context, drug evaluated, type and extent of patient-specific data incorporated, individualisation strategy (stepwise or multivariate), software platform, and model performance assessment methods. This information guided the organization of subsequent sections, in which individualization strategies are first described conceptually, followed by a synthesis of validation approaches, quantitative performance metrics, and clinical implications. Cross-referencing between text sections and summary tables was used to support a logical progression from study characteristics to performance evaluation and interpretation.

Artificial intelligence (AI)–assisted tools were used for the generation and editing of selected graphical elements.

## 3. Baseline PBPK Models: Structure, Inputs, and Softwares

The conceptual basis of PBPK modelling originates from early attempts to describe drug disposition at the whole-organism level, where tissues are interconnected through circulating blood. Nevertheless, the practical application of this mechanistic view was limited by the mathematical complexity of the resulting models and the lack of adequate computational tools [1]. With the advance of modern computing, PBPK models are now used to describe drug behaviour through the usage of physiological data and the inclusion of multiple compartments that represent individual tissues and organs, which are connected by blood flow. Despite the fact that these models can estimate standard pharmacokinetic parameters such as clearance, volume of distribution, and half-life of a drug, their structure also allows drug-specific information derived from in vitro or in vivo trials to be integrated for predicting plasma and tissue concentration-time profiles [19].

Within these models, each organ or tissue compartment is characterized by its physiological volume and is perfused by a certain blood flow rate [5]. Therefore, the inter-compartment drug transport is driven by organ-specific blood perfusion rates, allowing the model to capture how blood flow distributes the compound throughout the body [20]. Typical compartments in a PBPK model include all major organs involved in drug disposition, such as liver, kidneys, lungs, heart, brain, stomach, intestines, spleen, pancreas, muscle, adipose tissue, bone and skin [5]. In many cases, any remaining tissues that are not explicitly modelled are lumped together into a single “rest of the body” compartment to account for the rest of the body mass and blood flow [21].

Accurate model simulations require the specification of several key inputs. These include host-dependent and drug-dependent parameters which are discussed in the following subsections, as well as the software platforms that are commonly used for simulation.

### 3.1. Host-Dependent Inputs

Physiological parameters describing the organism are generally used as direct model inputs, reflecting prior knowledge of anatomy and physiology [5]. These physiological inputs include organ volumes, organ blood flow rates, cardiac output, tissue composition, abundance of relevant enzymes and renal/liver functional parameters (e.g., glomerular filtration rate, biliary excretion capacity, and overall hepatic functional status) [22]. All these properties vary depending on the species or population considered [5]. For instance, pediatric PBPK models incorporate age-dependent changes in physiological factors, such as in body weight, height, organ size, blood flow and interstitial and vascular space, to create a child-specific model [20]. Elderly populations and patients with diseases are handled similarly by adjusting physiological inputs [23,24].

In the late 1990s, Brown et al. described how PBPK modellers selected physiological parameters from the literature, relying on a limited set of default descriptions for organ volumes, blood flows, cardiac outputs, and tissue composition [25]. These values were mostly compiled from large anatomical and perfusion databases of healthy, resting, and non-anesthetized subjects and expressed primarily as fractions of the body weight or cardiac output with the explicit expectation that defaults would be replaced when study-specific data were available [25]. Contemporary PBPK models continue to represent the body as a series of physiological compartments defined by organ-specific volumes and blood flows [26]. These compartments are now further characterized by composition-based parameters that enable the mechanistic estimation of tissue-to-plasma partition coefficients (Kp). Modern tissue composition-based approaches, such as the Rodger and Rowland method, explicitly integrate drug physicochemical properties with organ composition to predict tissue distribution, and these methodologies are now routinely integrated within specialized PBPK software platforms [20,26,27].

While these parameters primarily describe the anatomical and hemodynamic structure of the organism, PBPK models also rely on biochemical and functional descriptions to capture drug-specific disposition processes. For orally administered drugs, both the gastric and intestinal mucosa contribute to first-pass metabolism. The fraction of drug that escapes goes to the liver for further processing and excretion [28]. In fact, the liver represents the principal organ for drug metabolism and elimination, as it contains the highest abundance of Phase I and Phase II metabolizing enzymes [29]. Importantly, cytochrome P450 (CYP)-mediated metabolism (Phase I) not only governs drug clearance but can also influence pharmacological efficacy, underscoring the dual role of these enzymes in both therapeutic and adverse drug responses. In PBPK models, major drug-metabolizing CYPs are represented, as these cover the bulk of small-molecule metabolism. For instance, members of the CYP1, CYP2 and CYP3 families are responsible for approximately 80% of drug metabolism [30]. Each CYP enzyme can be assigned a relative expression level across organs, with the liver exhibiting the highest overall CYP abundance and the most diverse CYP profile, the intestine showing a lower, but compositionally distinct CYP expression dominated by CYP3A isoforms, and the kidneys displaying a comparatively minor contribution to total CYP content. In addition to CYPs, Phase II metabolism enzymes, such as uridine diphosphate (UDP)-glucuronosyltransferases (UGTs), are incorporated, but differ, as they are more ubiquitously expressed across liver, intestine, and kidney [31].

PBPK models include parameters such as the enzyme’s intrinsic clearance (Cl_int_) or maximum metabolic rate (V_max_) and Michaelis–Mendis constant (K_m_). These parameters are commonly derived from in vitro studies using human liver microsomes, hepatocytes, or recombinantly expressed enzymes. Data from recombinant enzymes, when combined with information on hepatic enzyme abundance and variability, enables extrapolation of intrinsic clearance to the whole liver and population-level predictions in PBPK models [32].

Most software platforms implement population-based libraries in which enzyme abundance and activity vary across individuals according to demographic, physiological and genetic covariates [33]. A classic example is the polymorphism associated with CYP2D6. PBPK models can actually create virtual individuals who are poor, intermediate, extensive or ultrarapid metabolizers by scaling the Cl_int_ for CYP2D6 accordingly, often based on known activity score systems or allele frequency data [10]. Furthermore, age, weight, disease state, and induction by concomitant drugs are also handled by adjusting the relevant parameters. For instance, considering that smoking induces CYP1A2, a PBPK model of smokers might include higher activity of the enzyme [34]. Finally, marked interindividual variability in UGT abundance, particularly for UGT2B17, provides a mechanistic basis for population-level variability in Phase II metabolism within PBPK models [31]. An interesting strategy to address interindividual variability concerning CYP activity is the in vivo phenotyping approach using probe-drug cocktails that allow the concurrent assessment of several enzyme activities under identical physiological conditions [35]. Such phenotypic data has been proposed as a valuable input for PBPK modelling, particularly in studies aiming to individualize models to reflect subject-specific physiology and enzyme activity, thereby enabling the generation of VT [36,37].

Furthermore, transport proteins are membrane-bound pumps that control drug movement across cellular barriers, influencing drug absorption and clearance [38]. Contemporary PBPK models increasingly incorporate key transporters. These can be subdivided into two main categories: solute carriers (SLC) transporters, which primarily mediate drug uptake, including organic anionic transporting polypeptides (OATPs), organic anion transporters (OATs) and organic cation transporters (OCTs), and ATP-binding cassette (ABC) transporters, which function predominantly as efflux pumps, such as P-glycoprotein (Pgp) and Breast Cancer Resistance Protein (BCRP) [39]. In PBPK models, transporter processes are parameterized using kinetic constants, such as K_m_, V_max_ and Cl_int_. These parameters are typically derived from in vitro studies using transfected cell systems or membrane vesicles. To enable extrapolation to the in vivo setting, these results are then scaled to the human organ level by accounting for differences in transporter protein abundance between the experimental system and the target tissue, often informed by quantitative proteomics [40]. Historically, the routine application of PBPK modelling to CYP-mediated drug–drug interactions preceded its use for transporter-mediated drug–drug interactions, largely due to uncertainty in the predictive performance of transporter models. In fact, robust prediction of transporter effects remains challenging, as multiple uptake and efflux processes often occur at the same biological barriers [41]. Importantly, similar to drug-metabolizing enzymes, transporters exhibit substantial interindividual variability arising from genetic polymorphisms and differences in expression levels driven by age or disease [42,43,44]. A well-established example is the genetic polymorphism associated with OATP1B1 (SLCO1B1c.521T>C) which results in reduced function and can be represented in PBPK simulations. Such modelling approaches have successfully reproduced clinically observed alterations in drug exposure and internal concentration profiles by incorporating genotype-specific changes in transporter activity into hepatic uptake parameters, highlighting the utility of PBPK frameworks for integrating transporter variability into predictions of pharmacokinetics [42].

Collectively, interindividual variability in anatomical, physiological, biochemical, and functional determinants constitutes a major source of variability in drug pharmacokinetics and provides a key mechanistic rationale for the development of individualized PBPK models.

### 3.2. Drug-Dependent Inputs

The drug properties include all the parameters that are specific for the compound under study, including compound lipophilicity (logP, logD), solubility, molecular weight (MW), and pka values, all independently included without the need for any information about organisms’ physiology [26]. These drug-specific parameters are directly related to ADME, therefore enabling the simulation of the pharmacokinetics of a given compound. For example, lipophilicity and pka strongly influence solubility, membrane permeability and protein binding, thereby affecting pharmacokinetic parameters [45]. Accordingly, a range of physicochemical descriptors, including pka, MW, lopP, number of hydrogen bond donors and acceptors, and polar surface area, can be used to estimate ADME-related parameters [46]. In the following paragraph, each step of ADME will be briefly analyzed considering several of those drug-specific parameters.

For a drug to be absorbed, it must first be in solution [47]. However, compounds with high logP may present low solubility, limiting absorption [48]. In fact, studies indicate that an intermediate range of logP is associated with optimal bioavailability, whereas excessively high logP values are often linked to poor solubility and increased tissue accumulation, both of which compromise drug absorption [49,50]. Once in solution, drugs may cross biological membranes through several mechanisms, including passive diffusion, facilitated passive diffusion, active transport and pinocytosis [47]. In addition to logP, both pka and MW influence absorption, particularly through their effects on ionization state and membrane permeability [45,47]. Overall, it appears to be possible to simulate absorption in a relatively complete manner using drug-specific physicochemical parameters alone. Concerning distribution, intrinsic physicochemical properties of a drug largely determine its ability to distribute throughout the body. For instance, compounds with higher logP and lower solubility more readily penetrate lipid-rich tissues, resulting in a higher volume of distribution. In contrast, drugs with low logP tend to remain largely confined to the plasma and therefore exhibit a lower volume of distribution [51]. Metabolism and excretion are also influenced by these factors, including the clearance route, as it has been demonstrated that lipophilic compounds are more easily excreted by the liver, while hydrophilic drugs are normally excreted by the kidneys [52].

In summary, drug-dependent inputs provide the compound-specific mechanistic foundation upon which PBPK models simulate ADME processes. When integrated with host-dependent physiological, biochemical and genetic factors, these parameters may enable PBPK frameworks to move beyond population averages towards individualized predictions.

### 3.3. Software Platforms and Population Libraries

PBPK modelling is supported by a variety of commercial and open-source platforms, each with distinct libraries and features [26]. These softwares can be applied to anticipate the impact of food intake, assess formulation-related differences in drugs, elucidate mechanisms underlying low bioavailability for orally administered compounds, evaluate the influence of organ impairment and characterize potential drug–drug interactions [53]. Industry-leading commercial tools include GastroPlus^®^ (developed by Simulations Plus Inc., Lancaster, CA, USA) and Simcyp^®^ (Certara, Inc., Radnor, PA, USA), while prominent open-source packages include PK-Sim^®^/MoBi^®^ (Open Systems Pharmacology, Saterland, Germany) and other flexible frameworks [26].

GastroPlus^®^ was the first commercially available software for whole-body PBPK modelling, despite being originally designed for mechanistic absorption (ACAT model) [53]. Through modular extensions such as PBPKPlus and PEAR, the software supports tissue-level PBPK model construction, in vitro to in vivo extrapolation, interspecific extrapolation, and population-based physiology, including pediatric and region-specific demographic data [54]. Its strengths include absorption, dissolution, gut physiology, and formulation optimization, particularly for oral dose, bioavailability, and food-effect predictions [26].

Simcyp^®^ Simulator is a comprehensive, population-based PBPK engine widely used in drug development. It contains extensive physiological libraries and demographic databases. This platform offers specialized modules for target populations and the virtual populations available for use include multiple ethnicities and special groups, with curated meta-analyses of enzyme and transporter protein abundances in reference populations [54,55]. Virtual subjects in Simcyp are generated by correlated Monte Carlo sampling of covariates to preserve realistic relationships [55].

The open-source platform PK-Sim^®^ focuses on whole-body PBPK modelling for both human and animal pharmacokinetics, supporting cross-species extrapolation and use across early discovery to development [26]. It provides detailed anatomical and physiological models supported by extensive demographic libraries, covering multiple ethnic groups and special populations such as neonates and pregnant women. The database spans the full human lifespan, with age and weight-dependent physiological parameters and ontogeny functions that adjust metabolic and renal clearance [56].

**Figure 1:**
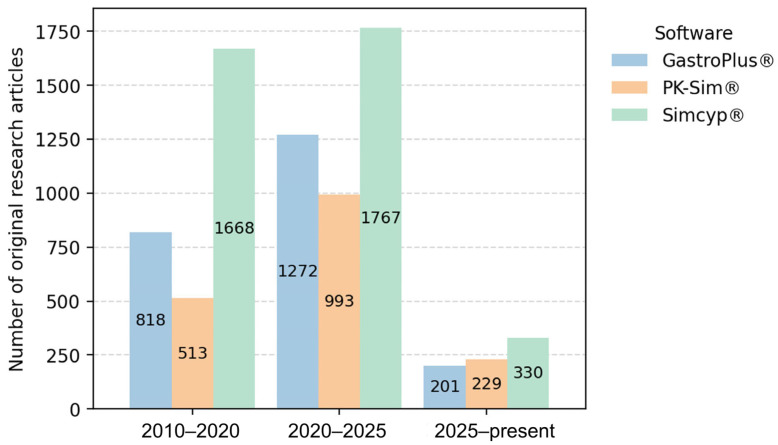
Temporal trends in the reported use of PBPK software platforms in scientific literature. The number of original research articles mentioning each software platform was estimated using Google Scholar searches (“software name” AND “PBPK”) across three time periods (2010–2020, 2020–2025, and 2025–present). Review articles were excluded. This search was performed on 6 January 2025.

Figure 1 illustrates the distribution of software platforms used in general PBPK publications, where Simcyp^®^ emerges as the most frequently applied tool. Similarly, among the studies focusing on individualized PBPK modelling and the generation of VTs that were selected for in-depth analysis (Section 5), Simcyp^®^ was also the most used platform, being employed in 8 out of the 11 reviewed studies [34,37,57,58,59,60,61,62]. Furthermore, Figure 1 shows that PK-Sim^®^ exhibits a modest upward publication trend, with slightly more articles published in 2025 compared with GastroPlus^®^. Notably, the remaining three individualized PBPK studies used PK-Sim^®^ [36,63,64], and, to the best of our knowledge, no studies to date have reported the use of GastroPlus^®^ for patient-specific PBPK modelling.

## 4. Conceptual Framework of PBPK-Based Virtual Twins

**Figure 2:**
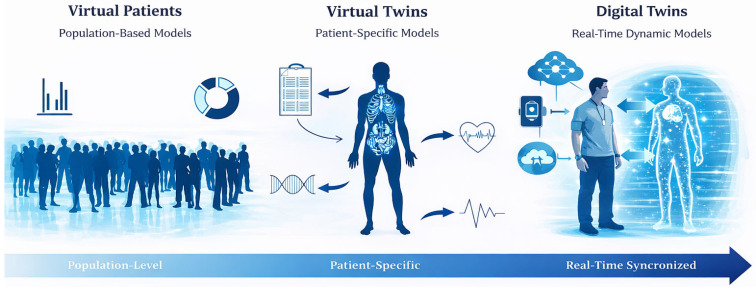
Conceptual distinction between virtual patients, virtual twins and digital twins in healthcare. Some graphical elements were generated and edited with assistance of artificial intelligence (AI) tools and were subsequently reviewed and adapted by the authors.

The increasing adoption of PBPK modelling within personalized medicine has fostered the development of conceptual frameworks that progressively shift from population-based representations toward individualized model-based approaches, enabling structured progression from precision medicine concepts to individualized clinical decision support. At the core of this shift lies MIPD, which leverages pharmacokinetic models to guide dosing decisions using patient data [14]. Building upon this principle, the notion of virtual patients allows the representation of PBPK through simulated individuals derived from population distributions [65,66]. A further conceptual step is achieved with VTs, where the model is explicitly tailored to reflect the demographic, physiological, phenotypic, and, when available, genotypic characteristics of a specific patient, thereby emphasizing individual-level prediction rather than cohort-based inference [16]. More recently, the concept of digital twins has emerged as an extension of virtual methodologies, proposing a dynamic and continuously updated representation of an individual that integrated real-world clinical data overtime [67]. Figure 2 schematically represents the distinction between these key concepts.

### 4.1. Precision Medicine and Model-Informed Precision Dosing (MIPD)

Precision medicine consists of translating up-to-date patient data into treatment decisions to maximize the long-term clinical benefit [68]. Through omics data, such as genomics, transcriptomics, proteomics, and others, this approach allows the prediction of risk and management of treatment for groups of patients with similar characteristics [69]. From this perspective, interpatient variability should not be perceived as statistical noise, but rather as a source of valuable information. Such heterogeneity can be leveraged to stratify patients and determine which treatments are most effective and when they should be administered. Accordingly, rigorous statistical methods should be applied to reliably extract and estimate optimal treatment strategies [68].

This field of medicine encompasses multiple domains, including drug development and pharmacotherapy. The concept of precision dosing was first introduced in the 1960s and has since evolved [70]. Nevertheless, at its core, it still aims to address the same problem: the substantial variability in pharmacokinetics, with great deviations in target therapeutic exposure, even in fixed-dose regimens [71]. Therefore, precision dosing allows drug therapy to be adjusted to a specific individual, guaranteeing that drug levels in the body reach and stay within a predefined target range that is both safe and effective [71,72].

With advances in computational and quantitative sciences, MIPD has emerged as a key precision medicine strategy. It leverages mathematical and mechanistic models of drug kinetics and dynamics together with individual patient data to choose the best dose. In practice, these approaches rely on models, which include PBPK models, calibrated on prior studies, to forecast how that patient will absorb, distribute and clear the drug [73]. While MIPD is generally framed within the paradigm of precision medicine, the focus of the present work lies in its application to personalized medicine. Specifically, by individualizing PBPK models through the integration of patient-specific demographic, physiological, phenotypic, and genotypic data, dosing decisions are tailored at the level of the individual patient rather than predefined subgroups.

### 4.2. Virtual Patients: Foundational Concepts in PBPK Modelling

Virtual patients are computer-based representations of hypothetical individuals drawn from a defined target population, constructed by sampling previously characterized physiological and biological parameters [65,66]. By randomly sampling known distributions of demographic and biological parameters, one can generate large cohorts of virtual patients that capture the spectrum of human variability [74]. In fact, the use of Monte Carlo simulation is a common approach to deal with interindividual variability and achieve optimal dosing in drug-related studies [75].

These virtual cohorts are subsequently employed in simulation experiments to systematically investigate drug behaviour across heterogenous populations [74]. In this framework, virtual patients enable in silico clinical trials that complement or partially replace animal and early-phase human studies, while allowing explicit modelling of population diversity. As a result, this approach can support safer and more effective dose selection, improve the prediction of efficacy and toxicity across subgroups, and ultimately contribute to reductions in morbidity and mortality [66].

### 4.3. Virtual Twins and Digital Twins in Individualized PBPK Modelling: Definitions and Conceptual Distinction

In the context of precision medicine, there is growing interest among researchers and clinicians in moving beyond traditional “one-size-fits-all” approaches to drug therapy [76]. Consequently, within PBPK modelling, increasing attention has been directed toward transitioning from population-based simulations to individualized implementations, in which patient-specific data are instantiated within mechanistic models, an approach often described as the construction of VTs [16,37]. Although the term digital twin is more widely used across engineering, healthcare systems, and information and technology literature [77], PBPK-focused studies most commonly adopt the term virtual twin to describe patient-specific model instantiations. Such individualized PBPK implementations remain relatively underexplored in the literature, with comparatively few studies fully adopting this paradigm.

In PBPK contexts, the term VT is commonly used to denote an individualized instantiation of a mechanistic human model parameterized with patient-specific characteristics and executed to generate predictions in a defined scenario. Notably, some healthcare authors describe the VT as dynamic, stating that it can be updated to reflect physiological evolution, new diagnoses, or pregnancy-related changes [78]. In contrast, within engineering and systems-medicine frameworks, the term digital twin is more consistently defined as a persistent cyber–physical representation that is coupled to its real-world counterpart through ongoing, and often bidirectional, data flows [79]. Under this interpretation, a digital twin is not a single model instance, but rather an integrated system-of-systems that enables continuous updating, monitoring, and decision support. Accordingly, a VT may be regarded as a component within a digital twin [80].

In this review, we therefore propose an operational distinction. PBPK VTs are typically model instantiation that require explicit re-simulation when patient data change, whereas digital twins denote an integrated system-of-systems in which model state is maintained and updated through automated data integration, such that updating is a property of the coupled pipeline rather than a manual rerun. We acknowledge that terminology remains inconsistent and that some publications use the terms interchangeably. Our proposed distinction (Figure 2) is intended to clarify model scope and updated mechanistic in PBPK-focused applications.

## 5. Individualisation of PBPK Models and Model Evolution

The individualisation of PBPK models has been explored through relatively diverse methodological pathways, reflecting different perspectives on how individual variability can be incorporated into model structure and parameterization. One of the earliest systematic attempts to address this challenge is provided by Polasek et al. (2018) [57], who presented a proof of concept for the use of VTs within PBPK contexts. Specifically, the authors addressed how models individualized using clinical data and TDM could be employed to predict exposure to the antipsychotic olanzapine, highlighting their potential to support MIPD [57].

Despite the author’s characterization of their work as the first of its kind, earlier PBPK studies had already incorporated individual patient data to individualize models, including a 2017 study on vancomycin pharmacokinetics in critically ill septic patients. However, the concept of “VTs” was not explicitly included in that work. Individualisation was primarily parameter-based and aimed at improving pharmacokinetic predictions by accounting for disease-related physiological changes [63].

Across the studies included in this review, a clear methodological distinction emerges, allowing the literature to be broadly divided into two categories: studies adopting stepwise individualisation approaches and those employing multivariate analysis from the outset (Table 1). Stepwise individualisation consists of progressively adding patient-specific parameters, with the aim of assessing how incremental personalization translates into improvements in model performance. Typically, initial steps rely on basic demographic information, followed by the inclusion of physiological parameters and, in later stages, phenotype or genotype-derived descriptors of drug metabolism and transport. This structured framework enables a transparent evaluation of how different categories of individual data contribute to improved pharmacokinetic prediction accuracy. In the vancomycin study in critically ill patients, progressive individualisation was used to disentangle the contribution of sepsis-related physiological changes and renal function to pharmacokinetic prediction accuracy [63]. A similar framework was adopted in a controlled caffeine study in healthy subjects, where successive inclusion of demographic, physiological, and phenotypic data enabled a quantitative assessment of how biomarkers, such as enzyme activity inferred from metabolic ratios, improved model performance [36]. In psychiatric settings, stepwise covariate virtualization was applied to patients treated with clozapine to evaluate the added value of environmental factors and pharmacogenetic information over basic demographic factors [34]. Finally, this methodology was extended to cardiovascular diseases and incremental incorporation of renal function and phenotypic markers of drug metabolism and transport that were used to individualize PBPK models for direct oral anticoagulants in patients with atrial fibrillation or thromboembolism [37].

Studies employing multivariate approaches from the outset appear to have been predominantly designed to address clinically complex scenarios in which multiple sources of interindividual variability coexist and interact. This strategy was first illustrated in the olanzapine study, where demographic, clinical, phenotypic, and genotypic information were simultaneously integrated to enable patient-level exposure prediction in a psychiatric setting [57]. Similar multivariate individualisation was applied in critically ill neonates receiving morphine, where physiology, dosing history, and genetic variation were considered to assess the predictive capacity of PBPK models in a highly vulnerable population [58]. Another study in neonatal settings used concurrent incorporation of pregnancy-related physiological changes and neonatal ontogeny to allow the exploration of risperidone and paliperidone pharmacokinetics using paired VTs (mother and neonate) [59]. More recently, this paradigm has been extended to increasingly data-rich scenarios, including the integration of environmental, pharmacogenetic, and molecular information to individualize exposure predictions for clozapine, midazolam, tacrolimus and amikacin across diverse clinical settings [60,61,62,64]. In the tacrolimus study, although a VT approach was applied to a single clinical case, predicted concentrations were not formally evaluated against observed clinical concentrations [62]. Moreover, among these studies, the 2024 study in the United States of America (USA) serves as a proof of concept for the use of liquid biopsy-derived molecular data to individualize PBPK models for midazolam exposure [61].

Collectively, these studies establish a new perspective in PBPK modelling contexts, demonstrating the potential of parameter individualisation, where the patient is represented as a distinct biological entity rather than as a member of a broader population in which substantial variability is still a challenge.

### 5.1. Methods to Assess Model Performance

The assessment of the performance of a statistical prediction model typically involves determining the extent to which predicted values approximate observed measurements and evaluating the overall agreement between predictions and observations [81]. Within individualized PBPK modelling contexts, several methods are used to assess model performance. The most common denominator across studies is the direct comparison between observed and predicted concentrations or pharmacokinetic parameters. Observed concentration values are typically obtained through blood sample analysis using liquid chromatography coupled with tandem mass spectrometry (LC-MS/MS) [34,36,37,57,59,60,61]. Alternatively, high-performance liquid chromatography (HPLC) has been used to quantify plasma drug concentrations [63], while in other studies the used methods were not addressed [58,64].

Model performance is commonly evaluated using goodness-of-fit (GoF) plots, which consist of graphical comparisons between observed and predicted concentrations, often displayed with an identity line, as well as residual-based plots. It should be noted, however, that in several of the studies these graphical diagnostics are presented under alternative terminology and are not always explicitly referred to as GoF plots, despite being methodologically equivalent [34,36,58,59,60,63,64]. In addition, alternative graphical approaches have been used, such as comparisons of predicted and observed frequency distributions of steady state through drug concentrations [57]. Furthermore, there is an article that assesses predictive performance using tables with comparisons of observed and predicted pharmacokinetic parameters [57], and others that use graphical summaries of these inputs [34,36,37].

To account for the bias (accuracy) of the individualized PBPK models, several metrics can be used. These include fold-based metrics, such as mean fold error (MFE) and geometric mean fold error (GMFE) [82], as well as percentage-based methods, such as percentage error (PE), mean prediction error (MPE), and median prediction error (MDPE), used to identify systematic over- or under-prediction [83,84].

Model precision was assessed less consistently across studies and the metrics typically used include root mean squared error (RMSE), absolute percentage error (APE), mean absolute prediction error (MAPE) and median absolute prediction error (MDAPE) [82,84].

Within the included studies, in addition to bias and precision metrics, model performance has also been evaluated using coverage-based criteria, such as the percentage of predicted concentrations falling within predefined fold-error limits (e.g., 1.25-fold or 2-fold) of the observed data or the requirement that the observed values fall within prediction intervals derived from VTs. Table 2 provides an overview of the performance evaluation methods used across the included individualized PBPK studies. Despite substantial heterogeneity in the specific metrics and criteria applied, the approaches can be broadly categorized into assessments of bias, precision, acceptance criteria, graphical representations, and statistical analysis.

### 5.2. Model Performance After Individualisation

#### 5.2.1. Models Following a Stepwise Approach for Individualisation

Individualisation of PBPK models generally improves the prediction of plasma drug concentrations, resulting in reduced bias and increased precision. Building on our prior strategy, we will first analyze studies that adopted a stepwise personalization approach. This progressive integration of patient-specific data, combined with performance assessment after each step, allows researchers to identify which specific additions contributed most to improvements in predictive accuracy.

Radke et al. (2017) [63] proposed a stepwise personalization strategy for a vancomycin PBPK model in patients with sepsis, in which progressively increasing amounts of physiological data were incorporated, resulting in models 1a, 1b and 2 (Table 1). In general, the incorporation of physiological data substantially improved prediction accuracy. Specifically, model 1a (without individualisation) showed a tendency toward overprediction, as reflected by positive MPE values, but still achieved better predictive performance than a model lacking sepsis-specific physiological adjustment. Among the individualisation steps that were evaluated, incorporation of creatinine clearance (model 1b) emerged as the most influential covariate, substantially improving predictive accuracy, with 88% observed concentrations falling within ±30% of the line of identity, with reduced bias and increased precision [63]. One plausible explanation for the pronounced improvement observed after incorporating creatinine clearance is the close physiological coupling between vancomycin elimination and renal function [85]. Just as vancomycin systemic clearance reflects the patient’s renal filtration capacity, creatinine clearance correspondingly reflects the individual’s ability to eliminate vancomycin, transforming it into a highly informative covariate for PBPK model individualisation. Moreover, despite the inclusion of additional patient-specific physiological information in model 2, only marginal differences were observed compared with model 1b, as indicated by the high Spearman correlation (r_s_ = 0.96) between the two models [63].

Furthermore, another study on the personalization of a PBPK model for caffeine with individual data derived from patients demonstrated that the addition of demographic data (step 1) improved the model in comparison with the baseline, with sex as a determinant factor for increased prediction ability. On the other hand, the individualisation of physiological data, including liver blood flow, glomerular filtration rate (GFR) and hematocrit on step 2, had limited impact. This was expected, as liver clearance of caffeine is somewhat limited [36]. These findings indicate that the choice of parameters to individualize should be made judiciously, considering the drug’s pharmacokinetic characteristics, patient-specific factors, and the clinical context. Step 3 proved to be the most impactful, as incorporating CYP1A2 phenotype led to a clear improvement in model performance, with noticeable impact on the area under the concentration-time curve (AUC). In the base model without individualisation, 45.83% of predictions fell within the 1.25-fold range, whereas in step 3, CYP1A2 personalization increased this value to 66.15%, further underscoring the critical importance of individualizing the activity of this enzyme. The present study goes a step further by reporting the number of patients for whom prediction accuracy improved with each model: 12 using the non-individualized model, 3 after step 1, 10 after step 2, and 23 after completion of all individualization steps [36]. This highlights the need to understand why more personalized and individualized models yield improved predictions for some individuals but not for others. One possible explanation is that the base model performs well for certain individuals but not for others. Although empirical Bayes estimation and shrinkage are not part of the PBPK framework used in this study, they provide a useful statistical analogy. When individual data are sparse or weakly informative, empirical Bayes estimates are pulled toward the population mean, and personalized predictions may perform worse than population-based predictions [86]. A similar principle may apply in PBPK modelling, so when individual input data are uncertain, noisy, or only weakly influential for drug’s pharmacokinetics, replacing population parameters with individual values can reduce predictive performance rather than improve it.

Additionally, in a distinct work by Mostafa et al. (2023) [34] the ability of VT personalization in improving the prediction of clozapine plasma concentrations was investigated. Clozapine is used in the treatment of schizophrenia and, although highly effective, has a narrow therapeutic window, making accurate dose adjustment an essential step. Its pharmacokinetics are strongly influenced by CYP1A2 activity, which is induced by environmental factors such as smoking, leading to lower plasma concentrations, while drugs such as ciprofloxacin inhibit it, resulting in higher concentrations. In this context, the authors showed that a low-covariate virtualization model, based only on demographic individualisation, performed poorly (R^2^ = 0.07) and systematically underpredicted clozapine concentrations. The addition of environmental data on the medium covariate virtualization model led to an improvement in prediction capacity, improving the R^2^ to 0.391, while maintaining a negative bias (Bland–Altman analysis indicated −138.48 ng/mL). Conversely, the high covariate virtualization model, whose main additional input was patient genetic information, did not lead to an improvement in R^2^. Nevertheless, it substantially improved systematic bias to −75 ng/mL, albeit remaining negative, indicating better accuracy despite unchanged overall explanatory power [34].

Finally, the last study employing a stepwise approach found that the individualisation of renal function (Step 2) predicted with improved accuracy the exposure to apixaban and rivaroxaban. Hence, creatinine clearance was a limiting factor for increased model performance [37]. There are descriptions in the literature that correlate creatinine clearance with apixaban clearance, so it would be expected that adding information about patient-specific renal function resulted in improved model prediction ability [87]. Contrary to what was reported in the previously mentioned studies, the inclusion of individualized phenotypic information did not lead to a significant improvement in model performance and was instead associated with overestimation of exposure. For example, incorporation of Pgp phenotype data resulted in MFE of AUC_tau_ of 1.31 for apixaban and 1.64 for rivaroxaban. Despite this lack of improvement in predictive performance, the addition of phenotypic data did enable identification of patients at the risk of bleeding, highlighting a distinct form of clinical utility [37]. This finding contrasts with previous studies using stepwise approaches, in which the inclusion of phenotypic information, particularly related to CYP enzyme activity, generally led to improved model performance [34,36]. Two main explanations can arise. First, the contribution of CYP3A activity may have been overestimated in the base PBPK model for apixaban and rivaroxaban, suggesting that this enzyme may not be the predominant determinant of these drug’s clearance. Second, unlike other enzymes discussed previously, such as CYP1A2, the relationship between phenotype and enzyme abundance for CYP3A may not be linear, potentially limiting the benefit of phenotype-to abundance conversion [37].

Taken together, these stepwise-approach studies underscore the importance of tailoring model personalization to the specific patient population and clinical context under study. Rather than focusing solely on the drug, effective model development requires careful consideration of patient characteristics and risk profiles to select the most informative personalization steps and achieve clinically meaningful outcomes. However, from a practical standpoint, any potential gain in predictive performance from personalized PBPK modelling must be carefully weighed against the clinal effort and patient effort to collect individual data, especially in cases when such data does not consistently translate into improved model accuracy across all populations [36].

#### 5.2.2. Models Following a Multivariate Approach from the Outset for Individualisation

Several studies have adopted a multivariate approach from the outset, incorporating multiple patient-specific variables simultaneously rather than through a predefined stepwise framework. Although some of these studies subsequently explored the relative contribution of individual covariates, personalization was not implemented through explicitly delineated sequential steps, but rather as an integrated multivariate process.

An early example is a 2018 Australian study that evaluated the prediction of olanzapine plasma concentrations in patients using individualized PBPK VTs. Following incorporation of patient-specific data, the individualized model achieved a relatively high predictive performance, with an R^2^ of 0.833 across 14 patients. Although genotype information was not included as part of a structured stepwise approach, separating patients by genotype further improved model performance, increasing R^2^ to 0.884, despite being in a smaller subset of seven genotyped individuals. Bland–Altman analysis revealed a modest systematic underprediction of approximately 10 µg/L prior to the next dose, but the model successfully identified an outlier patient with concentrations well below the therapeutic range [57]. This finding illustrated the potential clinical value of multivariate VT approaches, particularly for identifying patients that respond atypically and may therefore benefit most from individualized dosing strategies. Nevertheless, it is of the utmost importance to further explore these concepts in the light of pharmacodynamic behaviour to ensure clinical safety when applying such predictions. This concept was further extended in a distinct study on clozapine in patients with treatment-resistant schizophrenia, in which the authors moved beyond exposure prediction to explicitly dose selection required to achieve target plasma concentrations [60]. The model-based recommendations were considered appropriate in approximately 73% of patients. The individualized PBPK model demonstrated moderate predictive performance overall (R^2^ = 0.29), representing a follow-up application of a previously developed high-covariate personalization framework [34] to an independent patient cohort. Importantly, accounting for CYP1A2 induction in smokers substantially improved model performance, increasing R^2^ to 0.60. In contrast, overprediction was observed in patients receiving fluvoxamine, highlighting limitations in accurate representation of complex drug–drug interactions within individualized PBPK models [60].

Multivariate personalization has also been applied in pediatric settings. A study assessing morphine pharmacokinetics in critically ill neonates reported good overall concordance between predicted and observed plasma concentrations, with a median observed to predicted ratio of approximately 0.99. Overall, around 60% of predictions fell within a 2-fold of the observed values and 85% within a 3-fold range [58]. The study additionally evaluated the impact of haplotypes of the organic cation transporter 1 (OCT1), a key hepatic transporter involved in morphine uptake [88]. Model performance was superior in wild-type and heterozygous neonates compared with homozygous variants, suggesting that transporter-related variability remains an important determinant of predictive accuracy in this population [58]. Another study focused on constructing VTs for a pregnant woman and her newborn to predict paliperidone and risperidone concentrations. In the maternal model, all observed concentration values were within the 90% prediction interval both before and after delivery. Pharmacokinetics of the mother were primarily driven by increased CYP2D6 activity during pregnancy [59], consistent with literature describing hormonally and metabolically mediated alterations in bile-acid-dependent regulation of CYP2D6 [89]. The neonatal VT adequately captured the temporal evolution of paliperidone concentrations during the first days of life and renal function, particularly GFR, emerged as the determinant of paliperidone pharmacokinetics, underscoring the critical role of rapid postnatal renal maturation [59].

More recently, Rostami-Hodjegan et al. (2024) [61] provided proof-of-concept for integrating liquid biopsy data into PBPK model personalization and VT construction. By quantifying a broad panel of molecular biomarkers, the authors demonstrated substantial molecular variability even among patients with similar degrees of renal impairment. Incorporation of these data resulted in good predictive performance with slight overprediction, with an MFE of 1.38 and an MAFE of 1.78, as well as 76% and 92% of predictions of AUC within 2-fold and 3-fold of observed parameters, respectively. Notably, individualized incorporation of CYP3A-related variability appeared to exert the largest impact on reducing interindividual variability [61].

A further study addressed tacrolimus exposure during continuous intravenous (IV) infusion and IV-to-oral switching in transplant recipients using PBPK simulations supported by the generation of a single VT, based on a clinical case. However, the VT was applied retrospectively and exploratorily, rather than as a formal predictive model. The primary outcome was to demonstrate that incorporating individual patient characteristics shifts PBPK simulations away from population-average predictions toward values more consistent with the specific clinical context. Therefore, the study did not quantitatively assess improvements in predictive performance attributable to VT personalization [62].

Finally, a multivariate PBPK model developed for amikacin in patients with sepsis demonstrated robust predictive performance, with 83.8% of predictions within two-fold errors and 45% within a 1.25-fold range. While the authors supported the use of VT as a complementary decision-support tool, they emphasized that such approaches should not replace TDM, given the persistence of unexplained interindividual variability [64].

## 6. Limitations and Future Perspectives

Considering individualisation of PBPK models is a slightly novel area of investigation, several methodological and conceptual limitations can be identified across the reviewed studies, which collectively constrain the robustness, interpretability, and clinical application of the proposed modelling approach. Nevertheless, advancements in hospital information systems and emerging areas within personalized medicine, such as digital twins, may have a role in mitigating some of these challenges.

A predominant limitation is the small sample size, which reduces statistical power and hampers extrapolation of obtained data to broader and clinically heterogeneous populations. However, not all studies explicitly acknowledge these limitations. In a subset of publications, limited sample size is recognized as a key constraint [36,57,63]. In multiple cases, specific subgroups were under expressed, such as rare genotypes or distinct physiological states, and some cases relied on single cases or very limited cohorts, further limiting generalization [58,59]. By contrast, at least one study explicitly defined its cohort size as more than sufficient, including approximately 100 patients per treatment drug [37], while another considered the available sample size sufficient for proof-of-concept purposes concerning the use of liquid biopsy data [61]. Nevertheless, through the literature, there appears to be no consistent or formally justified criteria for determining the number of participants required, with cohort sizes often reflecting data availability rather than prospective study design, resulting in substantial heterogeneity in sample size across studies.

Another major limitation relates to data incompleteness and limited experimental control. In fact, several studies implicitly or explicitly relied on retrospective clinical data, acknowledging limited control over key variables, such as dosing history, timing of sample collection, adherence, and comedications [57,61,64]. Although such datasets enable real-world investigations, they further complicate causal inference and generalizability, by further introducing uncertainty and potential bias.

Limitations in capturing interindividual or intraindividual variability were also reported. Many approaches assumed a fixed physiological state, even when disease trajectories were acknowledged to be heterogenous [63]. This limitation highlights the potential advantage of digital twin frameworks, which, as proposed in this review, allow continuous updating of individual models as new patient-specific data becomes available.

A further recurring issue concerns limitations in enzymatic and transporter phenotyping. In several studies, for phenotypic data to be included in the model, enzymatic activity was inferred indirectly using metabolite-to-drug ratios or phenotypic probes, which may lack specificity, be influenced by absorption variability, or not be generalizable across different drugs [36,37].

Beyond these pharmacokinetic-centered limitations, an important future perspective lies in the more systematic integration of pharmacodynamics to better assess drug response, safety, and toxicity. Individualized exposure predictions alone may be insufficient to inform clinical decision-making. Incorporating pharmacokinetic-pharmacodynamic relationships within individualized PBPK frameworks could therefore improve prediction of both therapeutic efficacy and adverse effects.

Digital twins may further address several of the limitations identified in this review. Unlike static individualized PBPK models, digital twins aim to provide a dynamic and holistic representation of an individual, with all systems integrated and a more accurate prediction of drug behaviour and patient response over time as new data becomes available.

Overall, while current approaches to PBPK model individualisation and generation of VTs remain constrained by several factors, the reviewed studies collectively demonstrate the conceptual feasibility and potential clinical relevance of this modelling paradigm. Attention should be given in future studies to the process of selection of the factors to be individualized, as these decisions should be guided by the specific cohort and clinical context, as well as by the capability and willingness to undertake additional sample collection and laboratory testing. Continued methodological refinement, broader and better-structured datasets, and closer integration of pharmacodynamic endpoints will be essential to strengthen model robustness and translation impact.

## 7. Discussion

PBPK models are powerful tools for hypothesis generation, allowing researchers to explore drug behaviour, test scenarios, and support experimental design while reducing costs and minimizing the reliance on animal studies. These models offer a complementary approach to what is currently applied in clinical practice, namely TDM, potentially allowing dosing decisions to be informed prospectively rather than empirically. Current applications are more often based on virtual patients and population-level approaches. While these methods attempt to capture variability, they rely heavily on average physiological representations and predefined assumptions, which can limit their ability to accurately reflect interindividual variability.

To address these limitations, VTs have emerged as a promising alternative. Unlike virtual patients, VTs incorporate patient-specific data to generate individualized predictions. This may enable relevant clinical applications, including dose individualization, identification of patients at risk of subtherapeutic exposure or toxicity, enrichment of clinical trials, and optimization of therapy in special populations such as critically ill patients, neonates, pregnant women, and individuals with organ impairment. Studies employing individualized PBPK models typically assess model performance by comparing predicted and observed concentration-time profiles using measures of bias and precision. Within this framework, two main strategies are commonly observed: stepwise individualisation, where parameters are added sequentially, and multivariate approaches, where multiple patient-specific inputs are incorporated from the outset.

Overall, the inclusion of individual patient data has been shown to improve model performance, although the magnitude and consistency of this improvement vary across drugs, populations, and clinical settings. Physiological parameters, particularly those related to renal function, consistently demonstrate substantial predictive value, reflecting the close coupling between kidney function and drug elimination. Demographic variables have also been shown to enhance predictive accuracy in certain studies. In contrast, the benefits of incorporating CYP phenotypic data are less consistent, likely reflecting both enzyme-specific differences and the indirect nature of commonly used phenotyping methodologies, which may not robustly reflect true enzymatic capacity. Similar ambiguities are observed for genotypic data, which in some cases significantly improves predictive performance, while in others primarily reduces bias without fully resolving prediction error. These findings highlight that no universal individualisation strategy is likely to be optimal. Instead, parameter selection should be guided by the pharmacokinetic properties of the drug, the characteristics of the patient population, and the clinical context. Importantly, individualized PBPK models have demonstrated value in identifying outlier patients who respond atypically to therapy and are not adequately represented by population-based predictions. This ability underscores their potential utility in individualized risk stratification and dose optimization.

Despite these promising results, several challenges limit the clinical translation of PBPK-based VTs. These include small sample sizes, limited data availability and insufficient consideration of disease heterogeneity and pharmacodynamic outcomes. In addition, most studies remain retrospective or proof-of-concept, and longitudinal validation across evolving disease states is scarce. Integration of real-time patient data and interoperability with clinical information systems are still largely conceptual, further constraining scalability and routine clinical implementation. Although regulatory agencies increasingly accept PBPK analyses in drug development, their use for individualized clinical decision-making requires additional validation, standardized reporting practices, and clearly defined performance thresholds. Future efforts should prioritize the development of robust digital and computational infrastructures, harmonized data standards, and standardized validation strategies to support the transition of PBPK VTs from research applications to routine clinical use. In this context, digital twin frameworks represent a logical extension of PBPK-based individualisation, enabling dynamic updating of patient-specific models through continuous integration of multisystem data and facilitating embedding within clinical workflows.

## 8. Conclusions

This review highlights the ongoing transition from population-based PBPK modelling toward individualized PBPK VTs as an important advance in personalized medicine. Beyond improved predictive accuracy, they provide a mechanistic framework to understand why patients differ in drug exposure, supporting a shift toward patient-centred pharmacotherapy.

Looking ahead, PBPK-based VTs have the potential to shift precision dosing from an episodic, retrospective practice to a proactive and increasingly closed-loop clinical paradigm. By combining mechanistic PBPK with patient data, VTs can support timely, individualized decisions in contexts where “average physiology” is clinically insufficient. Importantly, the next stage of translation should move beyond demonstrating pharmacokinetic agreement and establish clinical utility through prospective, workflow-embedded studies that quantify patient-relevant benefits (target attainment, toxicity reduction, and healthcare resource use), while adopting standardized reporting and uncertainty quantification to define actionable thresholds for safe implementation. In parallel, future developments should prioritize more advanced digital twin frameworks as a logical extension of PBPK-based individualisation, enabling continuous integration of multisystem data and dynamic updating of patient-specific models over time.

## Figures and Tables

**Table 1:** Characteristics of the individualized PBPK virtual twin studies that are included in this review.

Individualisation Approach	Year of Publication	Country	PBPK Software	Aim of the Study	Number of VTs	Patient-Specific Data	Reference
Stepwise	2017	Germany	PK-Sim^®^	Develop and evaluate a PBPK model incorporating sepsis-associated physiological alterations to more accurately predict vancomycin pharmacokinetics in critically ill patients	10	**Model 1a**: literature data—body composition, protein binding, haematocrit, CrCL, cardiac output, and organ blood flow. No individualisation. **Model 1b**: literature data—body composition, protein binding, haematocrit, cardiac output, and organ blood flow. Individual CrCL. **Model 2**: literature data—cardiac output, and organ blood flow. Individual body composition, protein binding, haematocrit and CrCL.	[63]
	2021	Germany	PK-Sim^®^	Systematically evaluate if the personalization of a PBPK model for caffeine with individual data improves pharmacokinetic prediction accuracy.	48	**Step 1**: Individual demographic data—age, sex, ethnicity, body weight and height. **Step 2**: Individual demographic and physiological data—liver blood flow (phase-contrast MRI), pulmonary blood flow, GFR (estimated using plasma cystatin C concentration) and haematocrit. **Step 3**: Individual demographic, physiological and phenotypic data—CYP activity inferred from the paraxanthine/caffeine ratio.	[36]
	2022	Australia	Simcyp^®^	Evaluate whether the systematic inclusion of demographic, environmental, and genetic covariates in pharmacokinetic models based on VTs improves the prediction of clozapine plasma concentrations.	42	**Low covariate virtualization**: individual demographic data—height, weight, age. **Medium covariate virtualization**: individual demographic and environmental data—concomitant CYP inducers and inhibitors (including smoking and medications). **High covariate virtualization**: individual demographic, environmental and genotype data (genotype-predicted phenotypes for CYP2D6, CYP2C19, and CYP2C9.)	[34]
	2025	Switzerland	Simcyp^®^	Evaluate the effects of DOACs in patients with atrial fibrillation or thromboembolism using a VT approach.	100 (apixaban) + 100 (rivaroxaban)	**Step 1**: Individual demographic data—age, sex, ethnicity, weight and height. **Step 2**: Individual demographic and physiological data—creatinine clearance (Cockcroft–Gault formula) **Step 3**: Individual demographic, physiological data and phenotypic data (part 1)—Pgp activity (Geneva cocktail) **Step 4**: Individual demographic, physiological data and phenotypic data (part 2)—CYP3A4/5 activity	[37]
Multivariate analysis	2018	Australia	Simcyp^®^	Develop and evaluate an individualized PBPK modelling framework based on VT to predict olanzapine exposure at the individual patient level.	14	Simultaneous inclusion of individual demographic (age, sex, ethnicity, height, weight), clinical (smoking and olanzapine dosage), phenotypic (CYP1A2 activity inferred from the paraxanthine/caffeine ratio) and genotypic (CYP2C8, only in 7 patients) data.	[57]
	2020	USA	Simcyp^®^	Assess whether individualized PBPK models using patient-specific data can accurately predict morphine plasma concentrations in critically ill neonates.	32	Simultaneous inclusion of postnatal age, sex, dosing history, OCT1 haplotype.	[58]
	2022	Japan	Simcyp^®^	Develop and apply a PBPK VT model to assess the impact of pregnancy and neonatal ontogeny on risperidone and paliperidone pharmacokinetics and to explore its utility for MIPD in pregnant women and neonates.	2 (pregnant woman and her neonate)	Mother: demographic data—age, sex, weight, height, ethnicity, therapeutic information—risperidone dose, dosing regimen, treatment duration, and gestational timing. Neonate: weight and length, postnatal age and serum creatinine (to estimate renal function).	[59]
	2023	Australia	Simcyp^®^	Apply the previously used high covariate virtualization [34] to a new set of patients with treatment-resistant schizophrenia treated with clozapine.	11	Simultaneous inclusion of individual demographic, environmental and genotype data (CYP).	[60]
	2024	USA	Simcyp^®^	Evaluate the feasibility of using liquid biopsy-derived data to individualize PBPK models for predicting midazolam exposure in patients with varying degrees of renal impairment.	25	Simultaneous inclusion of individual demographic data (age, sex, body mass index), renal function (estimated GFR and or creatinine clearance) and molecular data obtained from liquid biopsy (projected hepatic expression of enzymes and transporters relevant to midazolam pharmacokinetics—primarily CYP3A4 and CYP3A5—inferred from mRNA detected in plasma-derived exosomes).	[61]
	2024	Switzerland	Simcyp^®^	To provide PBPK-based guidance for interpreting tacrolimus concentrations during continuous intravenous infusion and IV to oral switching in transplant patients, supported by the construction of an individualized VT for a real clinical case.	1	Simultaneous inclusion of individual demographic data (age, sex, weight and height), haematocrit, hepatic function, CYP3A5 genotype and dosing regimen.	[62]
	2025	Brazil	PK-Sim^®^	Use VT to individualize amikacin dosing in critically ill oncology patients by integrating patient-specific data.	40	Simultaneous inclusion of individual demographic data (age, sex, weight and height), creatinine clearance (Cockcroft–Gault formula) and amikacin dosing regimen.	[64]

PBPK, physiologically based pharmacokinetic; CrCL, creatinine clearance; MRI, magnetic resonance imaging; GFR, glomerular filtration rate; CYP, cytochrome P450; VT, virtual twin; DOAC, direct oral anticoagulant; Pgp, P-glycoprotein; OCT1, organic cation transporter 1; MIPD, model-informed precision dosing; IV, intravenous. Bold text is used to indicate distinct model versions, sequential individualization steps, or levels of covariate virtualization as explicitly defined within the corresponding studies.

**Table 2:** Overview of model performance evaluation metrics used across the included studies.

Bias/Accuracy Metrics	Precision Metrics	Coverage/Acceptance Criteria	Graphical Representations	Statistical Tests/Agreement	Reference
PE, MPE	APE, MAPE	±30% deviation from identity line in observed vs. predicted concentration graphic	Observed–predicted concentration plot (GoF)	Spearman correlation (model comparison)	[63]
GMFE (good prediction when <2)	RMSE	% within 1.25 and 2-fold	Observed–predicted concentration plot (GoF), VPC	ND	[36]
ND	ND	ND	Bland–Altman plots, Observed–predicted concentration plot (GoF)	Bland–Altman	[34]
MFE (0.8–1.25) Deming regression	ND	% within 1.25 and 2-fold; CI 95% of mean PK parameters within ±20%	Observed vs. predicted AUCs, Bland–Altman plots	Bland–Altman	[37]
MFE (0.5–2.0)	ND	% within 2-fold	Bland–Altman plots	Bland–Altman Paired *t*-test; ANOVA with Tukey post hoc (evaluate the effect of including covariates)	[57]
MDPE	MDAPE	2-fold and 3-fold criteria	Observed–predicted concentration plot (GoF)	ND	[58]
ME	RMSE	Observed values within 90% prediction interval, 2-fold criteria	Observed–predicted concentration plot (GoF), VPC	ND	[59]
ND	ND	ND	Observed–predicted concentration plot (GoF)	ND	[60]
MFE	MAFE	2-fold and 3-fold criteria	Observed vs. predicted AUCs	ND	[61]
ND	ND	Observed values within 90% prediction interval, % within 1.25 and 2-fold	Observed–predicted concentration plot (GoF), VPC	ND	[64]

PE, percentage error; MPE, mean prediction error; APE, absolute percentage error; MAPE, mean absolute prediction error; GoF, goodness of fit; GMFE, geometric mean fold error; RMSE, root mean squared error; VPC, visual predictive check; ND, not described; MFE, mean fold error; CI, confidence interval; PK, pharmacokinetic; AUC, area under the concentration–time curve; ANOVA, analysis of variance; MDPE, median prediction error; MDAPE, median absolute prediction error; ME, mean error; MAFE, mean absolute fold error.

## Data Availability

No new data were created or analyzed in this study. Data sharing is not applicable to this article.

## Abbreviations

The following abbreviations are used in this manuscript:

TDM	Therapeutic Drug Monitoring
PBPK	Physiologically Based Pharmacokinetic
MIPD	Model-Informed Precision Dosing
QSP	Quantitative Systems Pharmacology
ADME	Absorption, Distribution, Metabolism and Excretion
FDA	Food and Drug Administration
VT	Virtual Twin
Kp	Tissue-to-Plasma Partition Coefficient
CYP	Cytochrome P450
UDP	Uridine Diphosphate
UGTs	UDP-Glucuronosyltransferases
Clint	Intrinsic Clearance
Vmax	Maximum Reaction Rate
Km	Michaelis–Menten Constant
SLC	Solute Carrier
OATPs	Organic Anion Transporting Polypeptides
OATs	Organic Anion Transporters
OCTs	Organic Cation Transporters
ABC	ATP-Binding Cassette
Pgp	P-Glycoprotein
BCRP	Breast Cancer Resistance Protein
logP	Logarithm of the Octanol/Water Partition Coefficient
logD	Logarithm of the pH-Dependent Distribution Coefficient
MW	Molecular Weight
USA	United States of America
LC-MS/MS	Liquid Chromatography–Tandem Mass Spectrometry
HPLC	High-Performance Liquid Chromatography
GoF	Goodness of Fit
MFE	Mean Fold Error
GMFE	Geometric Mean Fold Error
PE	Prediction Error
MPE	Mean Prediction Error
MDPE	Median Prediction Error
RMSE	Root Mean Square Error
APE	Absolute Prediction Error
MAPE	Mean Absolute Prediction Error
MDAPE	Median Absolute Prediction Error
GFR	Glomerular Filtration Rate
